# Therapeutic Applications of Curcumin Nanomedicine Formulations in Cardiovascular Diseases

**DOI:** 10.3390/jcm9030746

**Published:** 2020-03-10

**Authors:** Bahare Salehi, María L. Del Prado-Audelo, Hernán Cortés, Gerardo Leyva-Gómez, Zorica Stojanović-Radić, Yengkhom Disco Singh, Jayanta Kumar Patra, Gitishree Das, Natália Martins, Miquel Martorell, Marzieh Sharifi-Rad, William C. Cho, Javad Sharifi-Rad

**Affiliations:** 1 Student Research Committee, School of Medicine, Bam University of Medical Sciences, Bam 44340847, Iran; bahar.salehi007@gmail.com; 2 Departamento de Farmacia, Facultad de Química, Universidad Nacional Autónoma de México, Ciudad Universitaria, Circuito Exterior S/N, Del. Coyoacán, Mexico City 04510, Mexico; luisa.delpradoa@gmail.com (M.L.D.P.-A.); gerardoleyva@hotmail.com (G.L.-G.); 3 Laboratorio de Posgrado en Tecnología Farmacéutica, FES-Cuautitlán, Universidad Nacional Autónoma de México, Cuautitlán Izcalli 54740, Mexico; 4 Laboratorio de Medicina Genómica, Departamento de Genética, Instituto Nacional de Rehabilitación Luis Guillermo Ibarra Ibarra, Mexico City 14389, Mexico; hcortes_c@hotmail.com; 5 Department of Biology and Ecology, Faculty of Science and Mathematics, University of Niš, 18000 Niš, Serbia; zstojanovicradic@yahoo.com; 6 Department of Post-Harvest Technology, College of Horticulture and Forestry, Central Agricultural University, Pasighat 791102, Arunachal Pradesh, India; disco.iitg@gmail.com; 7 Research Institute of Biotechnology & Medical Converged Science, Dongguk University-Seoul, Goyangsi 10326, Korea; jkpatra.cet@gmail.com (J.K.P.); gitishreedas@gmail.com (G.D.); 8 Faculty of Medicine, University of Porto, 4200-319 Porto, Portugal; 9 Institute for Research and Innovation in Health (i3S), University of Porto, 4200-135 Porto, Portugal; 10 Department of Nutrition and Dietetics, Faculty of Pharmacy, University of Concepcion, Concepcion 4070386, Chile; 11 Unidad de Desarrollo Tecnológico, Universidad de Concepción UDT, Concepcion 4070386, Chile; 12 Research Department of Agronomy and Plant Breeding, Agricultural Research Institute, University of Zabol, Zabol 3585698613, Iran; marzieh.sharifirad@gmail.com; 13 Department of Clinical Oncology, Queen Elizabeth Hospital, 30 Gascoigne Road, Hong Kong, China; 14 Phytochemistry Research Center, Shahid Beheshti University of Medical Sciences, Tehran 1991953381, Iran

**Keywords:** curcumin, cardiovascular disease, nanomedicine, nanocurcumin, liposome, nanoformulation

## Abstract

Cardiovascular diseases (CVD) compromises a group of heart and blood vessels disorders with high impact on human health and wellbeing. Curcumin (CUR) have demonstrated beneficial effects on these group of diseases that represent a global burden with a prevalence that continues increasing progressively. Pre- and clinical studies have demonstrated the CUR effects in CVD through its anti-hypercholesterolemic and anti-atherosclerotic effects and its protective properties against cardiac ischemia and reperfusion. However, the CUR therapeutic limitation is its bioavailability. New CUR nanomedicine formulations are developed to solve this problem. The present article aims to discuss different studies and approaches looking into the promising role of nanotechnology-based drug delivery systems to deliver CUR and its derivatives in CVD treatment, with an emphasis on their formulation properties, experimental evidence, bioactivity, as well as challenges and opportunities in developing these systems.

## 1. Introduction

**Figure 1:**
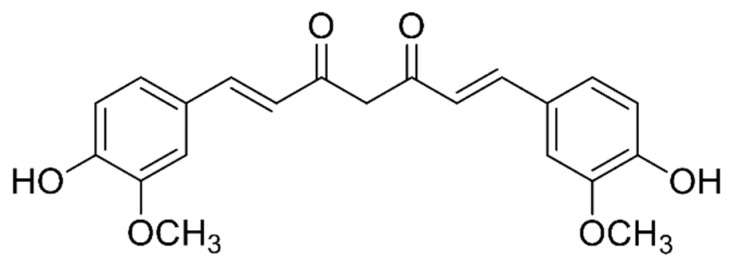
The chemical structure of curcumin.

Curcumin (1,7-bis[4-hydroxy-3- methoxyphenyl]-1,6-heptadiene-3,5-dione) is an active natural yellow colored polyphenol component that is found in *Curcuma longa* L. rhizomes (Figure 1). It is the main curcuminoid of turmeric (*C. longa*), a member of the Zingiberaceae family. It is widely used and sold as a food flavoring, herbal supplement, food coloring agent and even cosmetics ingredient with a history of usage that goes back to 1900 B.C. [1,2]. Formerly isolated in an impure form in 1815, Milobedeska and Lampe, in 1910, identified the chemical structure and chemically synthesized the compound [3,4,5]. Curcumin (CUR) commercial products have other CUR derivatives such as demethoxycurcumin and bisdemethoxycurcumin that has sometimes been studied instead of or beside CUR [6]. CUR is a bis-α,β-unsaturated β-diketone that shows keto-enol tautomerism. The enol form is predominant in alkaline medium while the keto form prevails in acidic and neutral pH [7]. This molecule has a unique chemical structure, anti-inflammatory, and antioxidant effects; it has been investigated and used in diverse fields, such as food, pharmaceutical, and textile industries [8].

Due to CUR chemical characteristics, it is considered to be a potent anti-inflammatory phytochemical that can interact with different inflammatory pathways that generated wide range pre-clinical and clinical therapeutic potentials for CUR [9,10]. In the past decade, a growing interest was noticed in CUR-based therapies in prophylaxis and treatment for different diseases, including CVD (atherosclerosis, diabetic cardiomyopathy, arrhythmia, hypertrophic cardiomyopathy, and heart failure) [11,12,13,14,15,16,17,18,19], cancer (colon cancer, breast cancer, and multiple myeloma) [20,21,22,23,24,25,26,27], neurodegenerative diseases (Parkinson’s, Alzheimer’s disease, and multiple sclerosis) [8,28,29,30], autoimmune diseases (osteoarthritis and rheumatoid arthritis) [31,32], psychological disorders [33,34,35,36,37], diabetes [38,39,40], pulmonary diseases [41,42,43], gastrointestinal disorders (gastric ulcers, indigestion, and dyspepsia) [44,45,46,47,48], ophthalmic disorders [49,50,51], and skin disorders [52,53,54].

A raising number of pre- and clinical studies have investigated CUR effects in CVD that is mainly put down to its antihyperlipidemic and anti-atherosclerotic properties [18]. In clinical trials, CUR was used in different doses ranging from 20–4000 mg with different effects on CV biochemical parameters [18]. CUR has protective properties against CVD through improving patients’ lipid profile, and it could be used alone or as a dietary adjunctive to conventional CV drugs [55]. CUR significantly increases beneficial serum parameters, such as apolipoprotein A (Apo A) and HDL, on the other hand, it reduces low-density lipoprotein (LDL), total cholesterol (TC), Apo B, plasma fibrinogen (PF), serum Cu/Zn, serum lipid peroxides (SLP), TC/HDL ratio, non-HDL, lipoprotein A (Lp(A)), serum pro-oxidant-antioxidant balance (PAB), and triglycerides (TG) [18]. 

CUR has been studied as a chemopreventive agent in atherosclerosis which is a chronic CVD that leads to the thick artery wall, it is shown that this effect is due to the reduction of SLP and TC serum levels, and the increase in HDL cholesterol [56]. Different studies have reported an improved lipid profile in patients with an acute coronary syndrome which is a situation that happens when the blood supply to the myocardium is blocked [10]. In some pre-clinical studies, CUR has shown efficacy and activity in heart failure treatment. This effect is attributable to inhibition of cardiomyocyte fibrosis, improvement in ventricular hypertrophy, and related-gene expression [57]. Other CUR cardio-protective potentials are used in myocardial ischemia/reperfusion injury, diabetic cardiomyopathy, arrhythmia, hypertrophic cardiomyopathy, and doxorubicin-related cardiotoxicity. The mechanisms suggested for these effects are attenuating apoptosis, oxidative stress, and inflammation [58].

Despite having enormous potential benefits, CUR has poor bioavailability that is attributed to its poor absorption, rapid metabolism, and high rate of systemic elimination from the body [59]. One of the major obstacles to deliver CUR is the poor solubility in aqueous media (estimated to be 3.21 mg/L at 25 °C, 0.4 µg/mL at the pH 7.4, and 11 ng/mL in aqueous buffer at pH 5), thus, 60%–70% of the orally administered drug is not absorbed and is excreted in feces [60,61,62]. On the other hand, CUR is soluble in ethanol, methanol, acetonitrile, chloroform, ethyl acetate, and dimethyl sulfoxide (DMSO) [63,64]. CUR has hydrophobic properties with an estimated octanol-water partition coefficient (log Kow) of 3.29 providing the molecule with good permeability capabilities in passing cellular membranes, however, these lipophilic properties diminish the oral absorption of CUR, thus, in biopharmaceutical classification system (BCS) it is considered to be a class II drug (low solubility and high permeability) [64,65,66]. Other barriers related to the stability of this molecule are high degradation rate and instability in body fluids because of rapid hydrolyzation at physiological pH. This molecule shows more stability in an acidic environment (pH range of 1.2–6.0) than alkaline media. The products that could found when CUR is degraded in hydrolytic conditions are diferuloylmethane, trans-6-(4′-hydroxy-3′-methoxyphenyl)-2,4-dioxo-5-hexenal, vanillin, ferulic acid, and ferulic aldehyde [67]. CUR instability is extended to light, exhibiting decomposition under UV/visible light exposure in both solid state and solution. The instability is considered to be a drawback for scale-up purposes in industrial point of view because of the minimized expected shelf-life [68]. CUR is rapidly metabolized through reduction or conjugation (sulfation or glucuronidation). Afterward, it extensively undergoes through systemic clearance from the body [69]. On the other hand, CUR is primarily eliminated in the bile, as hexahydrocurcumin glucuronides and tetrahydrocurcumin in intraperitoneally/intravenously administrations [7,70,71]. Pan, Huang and Lin [61] conducted a study on CUR tissue biodistribution after intraperitoneal administration in mice have also revealed low bioavailability of CUR vis this route. As a result, these studies show that despite the administration used route, CUR exhibits suboptimal blood concentrations and poor tissue biodistribution [72].

Nanomedicine is bridging the gap between pharmaceutical limitations and the therapeutic potentials of natural phytochemicals by improving the compound’s targeting, pharmacokinetics, efficacy, and cellular uptake [73,74,75,76,77,78,79,80]. Many studies have focused on CUR nanotechnology mediated drug delivery formulations in optimization the therapeutics uses of CUR for various diseases, such as cancer therapy [81,82,83,84,85,86,87,88,89,90], neurodegenerative disorders [91,92], wound healing [93], diabetes [94,95], and inflammatory diseases [96]. A wide variety of nanomedicine-based drug delivery systems are used to deliver CUR such as liposomes, polymeric nanoparticles, dendrimers, solid lipid nanoparticles, dendrosomes, nanogels, micelles, niosomes, cyclodextrin inclusion complexes, silver and gold nanoparticles, carbon nanotubes, nanoemulsions, nanosuspensions, exosomes, nanocrystals, and mesoporous silica nanoparticles. These promising platforms are also used for delivering CUR in tissue engineering [97]. This approach has emerged to face major drug delivery issues such as biodistribution limitations, rapid elimination, undesirable degradation/biotransformation, short half-life, and instability. Additionally, among different features provided by nanomedicine formulations are improving the solubility of hydrophobic drugs in water, the potentials of overcoming physiological barriers, increased permeability, offering the possibility of designing controlled release systems, and enhancing the circulation lifetime and pharmacokinetics [98,99,100,101,102,103]. One of the essential benefits that nano-mediated drug delivery could offer is the enhancement bioactivity and bioavailability through surface modifications, reduction of particle size, and entrapping CUR in within nanocarriers [104]. In the oral route, the bioavailability is enhanced by nanocarriers after improving solubility, protecting the drug from degradation in the gastrointestinal environment, and enhancing permeation in the small intestine; leading to an increase of drug levels in the blood stream [105]. Other emerging goals of nanocarriers is to achieve co-delivery of CUR with other drugs as an adjunct combinations therapy as an effective strategy to combat multi drug resistance [83,88]. Nanocarriers are also used to decrease the nonspecific drug uptake to undesirable tissues that leads to decreased toxicity [106,107]. Moreover, enhanced permeation and retention effect is one of the most important advantages of these systems which results in improving the circulation and accumulation of the loaded drug at the targeted sites, this means higher drug concentrations at the site of action which could help in minimizing the overall used dose and reduce adverse drug reactions [103]. Thus, leading to provision of agents and approaches specifically designed to improve CVD diagnosis and treatment [108,109].

In this present article, we aim to review and underline different studies and approaches looking into the promising role of nanotechnology-based drug delivery systems to deliver CUR and its derivatives in CVD treatment with emphasis on their formulation properties, experimental evidence, general bioactivity, and discussing the challenges and opportunities in developing these systems.

## 2. General Bioactivity of Curcumin in Cardiovascular Diseases

Studies showed very high cardioprotective potential of CUR including anticoagulant, anti-hypercholesterolemic and anti-atherosclerotic activity, as well as activities related to lowering the consequences of cardiac ischemia and reperfusion injury and regeneration of myocardium. Modes of action includes many molecular targets, including histone acetyltransferase (HAT-p300, involved in in the hypertrophy of cardiomyocytes), nuclear factor erythroid 2 (NFE2)-related factor 2 (NrF2, a major transcription factor involved in cellular redox homeostasis), NF-κB (nuclear factor kappa B, transcription factor upregulated in inflammatory/carcinogenic conditions), angiotensin II type receptor (AT1R, involved in cardiac hypertrophy), toll like receptor 4 (TLR4) and some other molecular targets such as SIRT3 and TGFβ/Smad-mediated signaling pathways.

### 2.1. In Vivo Studies

#### 2.1.1. Anti-Hypercholesterolemic Effect

Obesity is known as the main risk factor for CVD, whereas CUR presents a potent agent in its prevention through various mechanisms. This compound inhibits adipogenesis in 3T3-L1 adipocytes, angiogenesis and thus obesity, which was demonstrated in the study of Ejaz, et al. [110] on mice. This study demonstrated increased oxidation, decreased fatty acid esterification, reduced angiogenesis in an adipose tissue as well as reduced lipid metabolism in adipocytes, resulted in reduced total serum cholesterol.

Studies on rats fed with high fat diet (HFD) demonstrated that administration of CUR significantly reduced increase in body weight as well as levels of total lipids, TC and TG in comparison to the control group. Together with this, CUR intake reduced the high inflammatory response (tumor necrosis factor alpha and C-reactive protein, CRP) noticed in the control group, and alleviated total leucocytes, monocytes and lymphocytes, accompanied by decreased nitric oxide (NO) level in serum, aorta and cardiac tissue of the HFD-group [111]. 

Administration of CUR for 18 weeks lead to reduced early atherosclerotic lesions, lipid infiltration, intercellular adhesion molecule 1 (ICAM-1) and vascular cell adhesion molecule 1 (VCAM-1) localization together with decreased levels of plasma cholesterol, TG, LDL, Apo B levels as well as cholesteryl ester transfer protein (CETP) activity. In contrast to the above markers, plasma HDL and liver Apo A-I expression were increased. This study demonstrated anti-atherogenic efficacy of CUR comparable to those of lovastatin [112].

#### 2.1.2. Anti-Atherosclerotic Effect

Since CVD presents a condition highly induced by inflammatory response, CUR is a treatment of choice in their prevention due to high anti-inflammatory efficiency. Many studies have confirmed the efficacy of CUR in reducing the risk factors for atherosclerosis and CVD. For instance, a turmeric hydroalcoholic extract revealed to be protective against subcellular membranes lipoperoxidation [113], and damage of thoracic and abdominal aorta [114]. Similar findings were stated on LDL oxidation susceptibility and plasma lipid levels (cholesterol, phospholipid, and TG) [115]. However, mentioned studies used hydroalcoholic extracts of turmeric, while studies with pure CUR in this sense developed somewhat later. Olszanecki, et al. [116] administered CUR at a dose of only 0.3 mg/kg daily for four months to ApoE^−^/^−^ mice fed with HFD. The results pointed to efficiency of CUR intake in inhibition of atherosclerosis progression, but lack of impact to lipid levels (cholesterol and TG) or body weight in treated animals. The atheroprotective effect of dietary CUR (0.2% (w/w) for four months) in a mouse model of atherosclerosis was also studied together with its molecular and cellular targets at the vascular level [117]. It was found that CUR supplementation reduced atherosclerotic lesions up to 26%, together with decreased leukocyte adhesion and transendothelial migration via NF-κB-dependent pathways. The mentioned molecular mechanisms were related to increased expression of NF-κB protein inhibitor and decreased NF-κB/DNA binding and NF-κB transcriptional activity following tumor necrosis factor (TNF)-α induction, which were detected upon CUR exposure. In another study, CUR significantly ameliorated oxidized low-density lipoprotein (oxLDL)-induced cholesterol accumulation in macrophages and decreased the protein expression of scavenger receptor class A (SR-A) but increased that of ATP-binding cassette transporter (ABC) A1 [118]. Since CUR intake affected the expression of SR-A, ABCA1, ABCG1, and SR-BI in aortas and prevented atherosclerosis in apoE^−^/^−^ mice, it was proposed that inhibition of SR-A-mediated oxLDL uptake and promotion of ABCA1-dependent cholesterol efflux represent two crucial events in cholesterol accumulation suppression by CUR in macrophage foam cells transformation [118]. Similar reduction of oxLDL uptake has been reported in the study on LDLR^−^/^−^ mice after CUR administration at doses of 500–1500 mg/kg for 16 weeks [16], where inhibition of the fatty acid binding proteins aP2, together with decreased expression of cluster of differentiation 36 were detected in treated animals. In ApoE^−^/^−^ mice as model organisms, CUR (0.1% (w/w) for four months) was found to have another molecular target, toll-like receptor 4 (TLR4), whose downregulation leads to reduction in pro-inflammatory mediators and decreased atherogenesis [119]. Treatment of asthmatic ApoE^−^/^−^ mice with CUR (200 mg/kg/day, 8 weeks) demonstrated that CUR ameliorated the aggravation of atherosclerotic lesions and stabilized plaque by modulating the balance of T helper cell (Th)2 / regulatory T cells (Tregs) (Th2/Tregs) [120]. CUR intake markedly helped to normalize the elevated Th2 and Th17 cell numbers as well as Tregs in the spleen, while mRNA expression levels of M1 macrophage-related inflammatory factors (interleukin (IL)-6, IL-1β, and inducible nitric oxide synthase (iNOS)) also decreased in treated animals. 

CUR significantly affects vascular smooth muscle cells (VSMC), where it was found that it presents a potent inhibitor of platelet-derived growth factor (PDGF)-stimulated vascular cell functions, including migration, proliferation, collagen synthesis, and actin-cytoskeleton reorganization [121]. Also, CUR attenuated PDGF signal transduction and inhibited the binding of this growth factor to its receptors. The same study reported efficacy of CUR in attenuating neointima development in a rat arterial balloon-injury model [121]. Additionally, CUR inhibited oxLDL-induced cholesterol accumulation on rat VSMCs [122]. Other studies reported various effects of CUR to VSMCs, including inhibition of their proliferation [123], migration [124] and causing of the cytostatic effect at doses of only 5 µM [125]. These studies found that CUR stimulates the expression of caveolin-1 [122,123] and other molecular mechanisms such as blocking of NF-κB translocation and inhibition of matrix metalloproteinase (MMP-9) [124], or causing a cell cycle arrest by protein carbonylation, oxidative DNA damage and changes in the nucleolar activity, accompanied by elevated levels of p53 and p21 [125]. 

Parodi, et al. [126] demonstrated that orally administered CUR reduced proinflammatory cytokine expression in aortic wall, and decreased destruction of medial elastic fibers. In porcine coronary artery, CUR blocked endothelial disfunction induced by homocysteine [127], which was detected as inhibited epithelial nitric oxide synthase expression and superoxide anion production, as well as blocked vasorelaxation. Improvements of endothelial function by pretreatment with CUR were confirmed in human umbilical vein endothelial cells, where reduced permeability and monocyte adhesion were detected [117,128]. In the same model cells, CUR blocked NF-κB activation induced by TNF-α and reduced ROS, adhesion of monocytes, phosphorylation of c-Jun N-terminal kinase p38, and STAT3 [129]. Similar effect was reported for EA.hy926 endothelial cells where CUR reduced ROS levels and NADH activation [130]. In this study, when human microvascular endothelial cells (exposed to resistin) were treated with CUR, expression of P-selectin and fractalkine, intracellular ROS level and NADPH activation were reduced, as well as monocytes adhesion to HEC. Additionally, reduced oxidative damage has been found and related to JAK2/STAT3 pathway and suppressed apoptosis, which limited the reperfusion injury in myocardium when CUR was orally administered for 20 days [131]. Another study, performed on human microvascular endothelial cells, demonstrated protective effect of CUR against PM2.5-induced oxLDL-mediated vascular inflammation, where it reduced enhanced ROS, VCAM-1 and ICAM-1 expression levels [132].

CUR was also found to enhance the permeability of coronary artery via inhibition of several related protein expression, including MMP-9, CD40L, TNF-α, and CRP [133].

#### 2.1.3. Cardiac Ischemia and Reperfusion 

Heart ischemia can be reduced by CUR, which is confirmed by many scientific studies. In an in vivo model of thrombosis, CUR administration 30 min prior the ligation prevented ischemia-induced rise of malondialdehyde (MDA) contents and lactate dehydrogenase (LDH) release and also reduced decrease in heart rate and blood pressure following ischemia [134]. Assessing of oxidative stress-related biochemical parameters in rat myocardium following ischemia showed decreased levels of xanthine oxidase, superoxide anion, lipid peroxides (LPs) and myeloperoxidase and increased levels of SOD, CAT, GPx, and GST activities [135]. Postoperative elevation of plasma inflammatory cytokines IL-8, IL-10, cardiac troponin 1 and TNF-α was found in CUR-treated groups, related to NF-κB inhibition [136]. Beneficial effects of CUR (10 µM, 3 h prior stimulation) on cardiomyocytes were demonstrated to be related to toll-like receptor 2 and monocyte chemoattractant protein (MCP)-1 inhibition [137]. In the same study, cardiac ischemia/reperfusion model performed on rats fed with or without CUR (300 mg/kg/day; 7 days before and 14 days after I/R surgery) showed unchanged TLR2 in the infarct zone, decreased macrophage infiltration (CD68) and fibrosis, as well as recovered connexin 43 in the CUR-treated group. Preserving effect of CUR (applied only in the reperfusion period) on cardiac function after ischemia and reperfusion has also been confirmed in the study of Wang, et al. [138]. In the mentioned study, CUR reduced degradation of extracellular matrix and inhibited synthesis of collagen via TGFβ/Smad-mediated signaling pathway, which resulted with reduced extent of collagen-rich scar and increased mass of viable and functional myocardium [138]. In the study of Wang, et al. [139], effect of CUR (150 mg/kg/day, administered for 5 days prior ischemia) to early growth response (EGR-1), responsible for triggering inflammation-induced tissue injury after ischemia and reperfusion, has been investigated in rats subjected to 30-min ischemia and 180-min reperfusion. It was found that CUR significantly reduced expression of EGR-1 mRNA and protein, decreased inflammatory markers TNF-α, IL-6, p-selectin and ICAM-1, and reduced infarct size. Beneficial effects of CUR were demonstrated as reduced infarct size (2.5-fold), found in rats fed with CUR (10, 20, or 30 mg/kg/day) for 20 days and subjected to myocardial injuries by ligation of the coronary artery for 60 min [131]. Silent information regulator 3 (SIRT3) is a NAD-dependent histone deacetylase and has cardioprotective effects. Wang, et al. [140] investigated the role of SIRT3 signaling pathway in protective effects of CUR and found improved cardiac function and decreased infarct size via downregulation of the proapoptotic protein Bax and AcSOD2 and activation of SIRT3. 

Another very significant target for CVD prevention by CUR is its potential to prevent heart failure caused by hypertrophy of cardiomyocytes, which is a result of prolonged pressure or volume overload. It has been confirmed that CUR inhibits hypertrophic responses in cultured neonatal rat cardiomyocytes and that it prevents the deterioration of left ventricular (LV) systolic function, myocardial infarction and hypertensive heart disease [141], as well as diabetes-induced cardiac hypertrophy [142]. CUR is known to be a specific inhibitor of p300) [143], which regulates hypertrophy-responsive transcriptional factors and therefore presents a therapeutic agent for maladaptive hypertrophy of cardiomyocytes. This was demonstrated in several animal models of heart failure [141,142,144,145]. In combination with enalapril, CUR applied orally (50 mg/kg per day for 6 weeks) to rats after myocardial infarction enhanced LV fractional shortening (FS) and reduced cardiomyocyte diameter in the non-infarct area, as well as perivascular fibrosis [146]. Changes in gene expression (total of 179 genes), improved heart function, reduced infarct size and abnormalities in the activities of LDH and creatine kinase-MB (CK-MB) were detected in the group treated with CUR for only three days (75 mg/kg daily) [147]. The same authors proposed cytokine-cytokine receptor interaction, extracellular matrix-receptor interaction, focal adhesions and colorectal cancer pathway as those involved in the cardioprotective effects of CUR.

### 2.2. Clinical Studies

Many clinical trials confirmed beneficial effects of CUR in the prevention and treatment of various CV conditions. Cholesterol-lowering effect of CUR consumption has been demonstrated in many studies, where healthy subjects [12,14,56] or participants with hypercholesterolemia [15,148] consumed CUR for defined period of time (per os), ranging from 7 days to 6 months. It was found that when healthy subjects consumed CUR in doses from 80–4000 mg/day, positive effect on blood lipid profiles were observed and included decrease in SLP, lowering in TC [12,14,56] and LDL cholesterol, increase in HDL and Apo B [56,148]. In addition to enhanced lipid profile, DiSilvestro, et al. [14] found lowering of TC, plasma sICAM readings, plasma ALT activities and β-amyloid proteins by CUR treatment; the results for the same subjects demonstrated an increase of salivary radical scavenging capacities, plasma CAT activities, myeloperoxidase as well as plasma NO.

CUR was also found to be beneficial in the clinical trials performed on patients suffering from obesity [149,150,151], metabolic syndrome [152,153,154] or acute coronary disease [11]. When obese individuals consumed capsules of CUR (500 mg C3 Complex (curcuminoids formula + 5 mg bioperine)) for 30 days, serum TG were decreased, as well as LDL, TC/LDL ratio, and PAB in serum [14,149,150], together with increased Zn/Cu and a reduction in Cu/Zn ratio in serum [151]. Improved lipid status was also recorded in patients suffering from metabolic syndrome, where intake of CUR extract capsules (12 weeks) or C3 Complex (8 weeks) resulted with elevated HDL concentrations and reduced LDL, TG, TC/HDL ratio, non-HDL, TC, TG, and Lp(A) [152,153]. When healthy subjects positive for metabolic syndrome consumed either black seeds, either turmeric or their combination, it was found that turmeric alone improved body mass index, waist circumference and body fat percentage (BF%) after 4 weeks and reduced LDL and CRP following 8 weeks of consummation. In combination with black seeds, reduced BF%, TC, TG, LDL, CRP, and raised HDL were recorded in the serum of the participants [154]. Similar positive effects were found in clinical trials (3 studies: 1) *n* = 75; 2) *n* = 240 and 3) *n* = 70) on hypocholesterolemic patients, those with type II diabetes and acute coronary syndrome [11,13,15].

## 3. Nanomedicine: Nanoformulation and Cardiovascular Effects

Therapeutic application of most of the drugs reduces their viability and potential due to use of conventional phytochemical methods [107]. More than 40% of new drugs entities has shown slow absorption rate due to poor water solubility [155,156]. As a consequence of this, most of the newly-discovered drugs have low bioavailability and inefficacy in terms of its actions. A major challenge lies in improving the poor absorption of conventional drugs by finding perfect formulation without altering the physicochemical properties of drugs and targeted delivery. Nanoformulation is a unique drug package where a drug is encapsulated with nanoparticle to tackle the challenges of poor absorption, low bioavailability and inefficacy site specific delivery of drugs. In recent years, the uses of nanoformulated drugs are prominently increasing to enhance the therapeutic value of drugs [157]. These nanoformulated drugs possess certain features like quantum size effects, targeted size delivery, target specific, high surface to mass ratio, high solubility, and absorption [158]. 

Nanomedicine is a new area which is growing very fast in combination with nanotechnology and pharmaceutical sciences [159,160,161]. Nanoparticle possesses different characters including pharmacokinetic, efficacy, safety, and target specificity. This impart character is being exploited by the pharmaceutical researcher to include in drug formulation [159,160,161,162]. Some nanomedicines are under clinical trials for wide application and indications [160]. However, many challenges like better characterization, issue related to toxicity, regulatory guidelines, cost-effective and health care warnings are faced by the nanopharmaceuticals. 

### 3.1. Nanoformulations Characteristics

Nanoformulation size ranges from 10–100 nm in diameter [163]. Drugs are attached to the nanocarrier. Nanoformulation has certain properties while formulating nanodrugs. The nanoformulation must facilitate the drugs to timely reach site of action from the site of administrations. The site of administration may be through oral or injection as fluid. The formulation should also protect the drugs from detrimental effects of bodily environmental factors (pH, enzymes, temperature). It is being reported that preparation techniques of nanodrugs plays a great role in maintaining the desired characters for delivering the drugs at targeted area [164,165]. Nanoformulation may have nanospheres or nanocapsules types depend on method of preparation. In nanocapsules, drugs are embedded inside the cavity of polymer matrix, whereas drug is uniformly dispersed in nanospheres. Nanocapsules have larger size, higher degree of polymerization than nanosphere. In freeze drying technology, nanospheres can be easily lyophilized than nanocapsules due to its structure. 

### 3.2. Nanoformulation Techniques

The preparation of nanoparticles with CUR is a well-established method by several authors taking advantage of the liposolubility of the drug to incorporate into the internal phase of emulsions. The solubility of approximately 6 μg/mL guarantees high encapsulation efficiency in most cases. The use of organic solvents such as acetone and ethyl acetate for the internal phase of emulsions allows rapid solubility of the drug and subsequent solvent removal [91]. The method most frequently reported in the literature for the elaboration of nanoparticles with CUR is nanoprecipitation [166]. Briefly, the drug and the nanoparticle polymer are solubilized in an organic solvent at room temperature with moderate magnetic stirring, then the stabilizer is solubilized in water in a concentration range that can range from 0.5% to 5% w/v. Subsequently, the organic phase is poured into the aqueous phase, the change in solubility of the polymer and the drug results in the formation of the nanoparticles by the presence of the stabilizer and magnetic stirring [167]. Finally, the system is subjected to reduced pressure to remove the solvent, subsequent steps include purification of the formulation usually by centrifugation and then conditioning by lyophilization.

The most commonly used polymer is poly (lactic-co-glycolic acid) (PLGA), an excipient employed in the medical area for a long time and approved by the U.S. Food and Drug Administration (FDA). This polymer is biodegradable and its by-products enter the Krebs cycle [91]. Although the by-products slightly acidify the environment where it is degraded, there are few studies that indicate severe complications from this involvement. The biodegradation time, or in other words, the control of the release time, can be manipulated by the proportion of lactic:glycolic monomers. Even today it is possible to acquire several derivatizations by the main commercial suppliers that allow a higher vectorization. 

Moreover, for CV effects the option of PLGA-polyethylene glycol (PEG) is an attractive alternative due to the increase in the hydrophilicity of the nanoparticle corona, decrease in protein adsorption, increase in circulation time, and therefore, the possibility of reaching heart. The stabilizers most commonly employed in the nanoprecipitation technique for the formulation of nanoparticles with CUR include Tween 80, polyvinyl alcohol (PVA), and poloxamer 407 and 188. In general, PVA offers high stability with zeta potential values, usually greater than –20 mV and allows adequate reproducibility, while Tween 80 and poloxamers can offer a type of biological interaction that improves the therapeutic effect of the formulation. The choice of stabilizer usually consists of a balance of stability/therapeutic effect.

Certainly, the spontaneous shock of solubility when the organic phase is poured into the aqueous phase can cause a decrease in the reproducibility of the particle size, polydispersity index (PDI) and the efficiency of drug loading in the nanoparticles. However, it is a practical and fast method, even the most feasible for its industrial escalation. Today, it is possible to find in the pharmaceutical industries the appropriate instrumentation to produce PLGA nanoparticles in batches with an average capacity of 10 L or more.

An alternative to increase the reproducibility of CUR nanoparticles and drug loading efficiency is through the use of the emulsification-diffusion method [91,99]. This method also consists in the formation of an emulsion, but with the difference of the previous saturation of both phases. It also involves adding a quantity of water at the end. In the intermediate step of the emulsion, the addition of an additional fraction of water breaks the emulsion, the internal phase moves outward and causes precipitation of the polymer and drug due to the effect of the stabilizer and agitation [168]. The volume of the batch is greater than that obtained by nanoprecipitation and therefore the nanoparticles are in a lower concentration. The production time is longer than by nanoprecipitation and the stirring speed is greater than 1,500 rpm. In general, the same excipients can be used for both methods.

The first quality tests of CUR nanoparticles should consist of measuring the average particle size, PDI, zeta potential and morphology. These inspections will allow establishing parameters for the subsequent validation of the manufacturing method. The average reported size for CUR nanoparticles ranges from 100 to 200 nm, with a PDI value that reaches 0.05 in the best case, the zeta potential fluctuates depending on the type and concentration of stabilizer. The morphology by scanning electron microscopy or atomic force microscopy is usually spherical.

### 3.3. Types of Nanoformulation

In every year, new nanodrugs entered clinical investigation and some more are under pipelines in the very early stages. However, it is confirmed that nanodrugs are developing very fast beyond the expectation. The clinically trials nanodrugs has steadily increased since 2007 [159]. Selected nanoformulated drugs are listed in the following section.

#### 3.3.1. Liposomes Nanoformulation

Liposome is a spherical vesicle made from lipid bilayer membrane having a designed of empty core structure. Due to their unique properties, liposomes can be used in nanodrug formulations by encapsulating with nanoparticles. Liposomes were firstly identified as simple drug delivery system in 1970s [159]. Their size is 90 to 150 nm in diameter and is capable of self-assembling the hydrophilic or hydrophobic therapies into its empty core [159,160]. Hydrophilic drugs such as ampicillin and, 5-fuoro-deoxyuridine can easily fit into the empty core region of liposomes without any modification in drug ratio. Hydrophobic drug such as Amphotericin B and Indomethacin attached to acyl hydrocarbon chain of the liposomes rather confining to the empty core [169]. Liposomes are considered as one of the most viable drug delivery vehicles due to their specific designed in membrane structure that can facilitate incorporation of different types of drugs in them [170]. Their structural designed enable them to carry biomolecules such as monoclonal antibodies and antigens as conjugated ligands on its surface. Liposome based nanodrugs has advantages of extended retention time period in bloodstream providing longer time for treatment as compared to nonliposomal drugs. They are very much effective at the site of tumor infection area as they can accumulate the drug and deliver to the targeted cell. 

Liposomes can be divided into four types [171]: (1) conventional type liposomes, (2) PEG types, (3) ligan-targeted types, and (4) theranostic types. Drug loading in liposomes is not affected by the types of liposomes however it can be performed in two different ways, i.e., active (drug loaded after liposomes is formed) and passive (drug loaded during the liposome formation). Mechanical and solvent dispersion methods of passive loading are usually performed. 

Liposomal nanoformulations for drug delivery have been significantly increasing in pharmacology. These formulations have benefitted from the stability, biodistribution on those drugs having bioavailability or high toxicity [172,173,174,175]. While treating liposome as alone in intravenous blood stream, it got cleared by immune system due to having short half-lives [172]. However, nanoformulation on liposome helps it to minimize the clearance as PEG attachment protects it from easily accessible.

#### 3.3.2. Nanoformulation of Polymer

Polymer based nanoformulations are widely used in nanomedical research due to their unique properties such as easily synthesized, safety, and efficacy in delivery. Among the polymer, the most well-established polymer is PEG. Polymer based nanoparticle may have different size of single polymer chain (can be directly used as a therapeutic) or as a modifying agent. Polymeric nanoparticle can withstand the drug in the body for weeks and this character made it a promising carrier for numerous medications including cancer, diabetes, and vaccinations [161]. Polymer nanodrugs have following benefits over the conventional polymers: (1) Biodegradable, (2) longer retention time, (3) biocompatibility, and (4) solubility.

#### 3.3.3. Nanocrystals

Nanocrystals are solid drug particles of sizes within a 1000 nm range. They act as drug molecules without attaching any kind of carriers on their surface [171]. Nanocrystals possess peculiar type of characters such as increase saturation solubility, increased dissolution velocity and increased glueyness on the membrane surface. These characters allowed them to be one of the most promising molecules for nanodrugs. Nanocrystals can be made in two different ways: Top-down process and bottom-up process. Top-down techniques are based on size reduction from a relatively large molecule into smaller particles. Bottom-up techniques consist of smaller molecules to form individual large molecule. Bottom-up approaches for nanocrystals are commonly called as ‘precipitation methods. Bottom-up techniques includes: (1) Hot melt method, (2) solvent evaporation method, (3) hydrosol, (4) gas anti-solvent recrystallization, (5) rapid expansion of supercritical solutions, (6) and controlled crystallization during free-drying. Top-down techniques includes: (1) sono-crystallization, (2) precipitation, (3) high gravity-controlled precipitation technology, (4) multi-inlet vortex mixing techniques, and (5) limited impinging liquid jet precipitation techniques. Among all of the methods, precipitation is the most common method for production of nanocrystals. In nanosuspension nanocrystals, the dispersing medium are usually replaced by water or any aqueous media (liquid PEG and oils) [176,177]. 

Nanocrystals are versatile in nature, can be used to improve the pharmacokinetics or pharmacodynamics properties of organic or inorganic materials with poor solubility and bioavailability [178,179]. They possess a narrow, symmetric emission spectrum, tunable, and photochemical stability. They have optically active core region surrounded by a shell that provides a protective against the external environment, making them to less sensitive to photo-oxidation and medium changes. Nanocrystal promotes saturation solubility which can trigger on diffusion-based mass transfer across the biological membrane.

### 3.4. Curcumin Based Biocomposite Formulation

**Figure 2:**
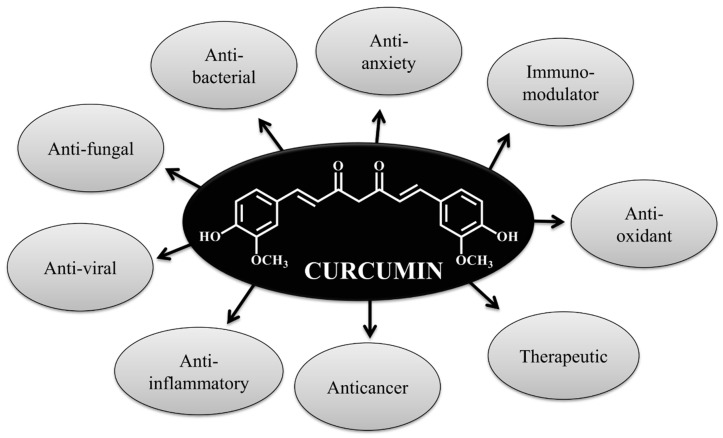
Different properties of curcumin.

CUR is a natural compound having diverse properties (Figure 2) in relation to therapeutic, antineoplastic, anti-microbial, anticancer and in treating of several pathologies, neurodegenerative, inflammatory and CVD [180] and it is a good molecule that can be used as encapsulating material with other biomolecules. CUR is a promising natural photosensitizer used in photodynamic therapy [181]. CUR can be formulated with nano-emulsified materials in treating breast adenocarcinoma cell line. This biocomposite is a well-designed drug delivery system which can exploit photodynamic property as therapeutic tools in an in vitro breast cancer model, MCF-7 cells [181]. CUR-nanoemulsion composite fulfill all the requirements to be an excellent drug delivery system.

In very interestingly, CUR can also form a composite with CaCO_3_ based solid dispersion formulation to enhance the dissolution rate of water [182]. This formulation was carried out using ethanolic CaCl_2_ as solution medium thereby diffusing the CO_2_. The interaction between CaCO_3_ and CUR helps 100% drug entrapment. This can be a novel solid dispersion preparation pathway for preparing oral administrated water insoluble drugs. 

CUR solubilizer can be checked for its solubility by formulation with other molecules. In a series of research findings, using non-linear quantitative structure–activity relationship (QSPR model) model, CUR solubility was found to be highest in composite formation with co-crystallized with pyrogallol [183]. 

In another effort, for treating peptic ulcer, CUR is formulated with low density material such as polypropylene foam powder, oils and various solubilizers. This biocomposite has prolonged gastro-retention time and improve insufficient CUR release [184]. This composite is a promising carrier for drug targeted at stomach using CUR formulation. 

**Figure 3:**
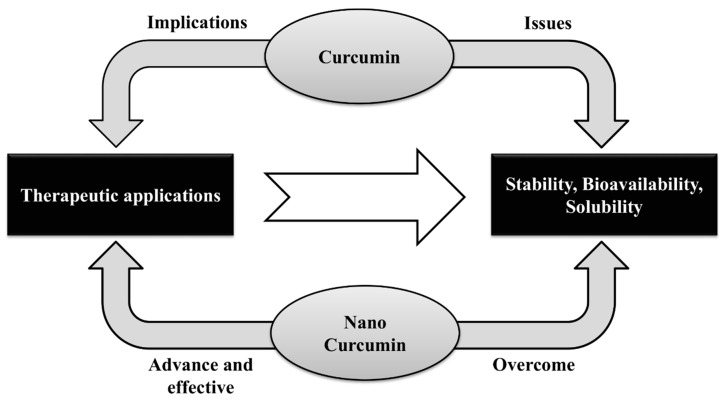
Curcumin and nanocurcumin.

The therapeutic potential of CUR has certain limitation in regards to poor solubility, bioavailability (Figure 3), and photostability. Onoue, et al. [185] reported to design and develop efficacious formulation of CUR with nanocrystal solid dispersion, amorphous solid dispersion, and nanoemulsion to overcome limitation of CUR formulation. These CUR-based formulations have improved physicochemical and pharmacokinetic properties.

CUR loaded nanoparticle drug delivery system plays a major role targeted delivery of drugs. Many CUR-based nanoformulations have been developed in order to ensure site specific target of cancer cells. CUR nanoparticulate is comparatively more effective than free CUR while tested against different cancer cell lines under in vitro conditions [186]. As part of the studies, this CUR-based nanocomposite showed longer half–life than free CUR while tested on mice. They have reported that, CUR nanoparticulate will have more potential as anticancer drug for treatment of malignant tumors than normal CUR treatment.

### 3.5. Preparative Methods of Nanocurcumin Formulation

CUR nanoparticles are usually prepared in different ways such as (1) coacervation techniques (polymer is directly allowed to dissolve in organic solvent, e.g., ethyl acetate and CUR is suspended directly in the solution and it is allowed to homogenize. Nanoformulation is collected after centrifugation) [187], (2) nanoprecipitation (also called as solvent displacement methods, polymer and CUR is allowed to suspends together to form drug- polymeric solution and then water is added under continuous stirring which results to form precipitation. Solution is then dried by evaporation) [188], (3) spray drying technology (A CUR nano-suspension having drug concentration of 10% (w/w) is dried using mini spray dryer. The spray dried nanocrystal is directly collected after the process is over) [189], (4) single emulsion techniques (It is a conventional method in which CUR nano-suspension are dispersed in a suitable solvent followed by homogenization or ultrasonification [190], and (5) wet milling method (nano-CUR is suspended in a suitable dispersing solvent followed by ultrasonification. The obtained CUR nanoparticle is collected by centrifugation [191]. 

**Figure 4:**
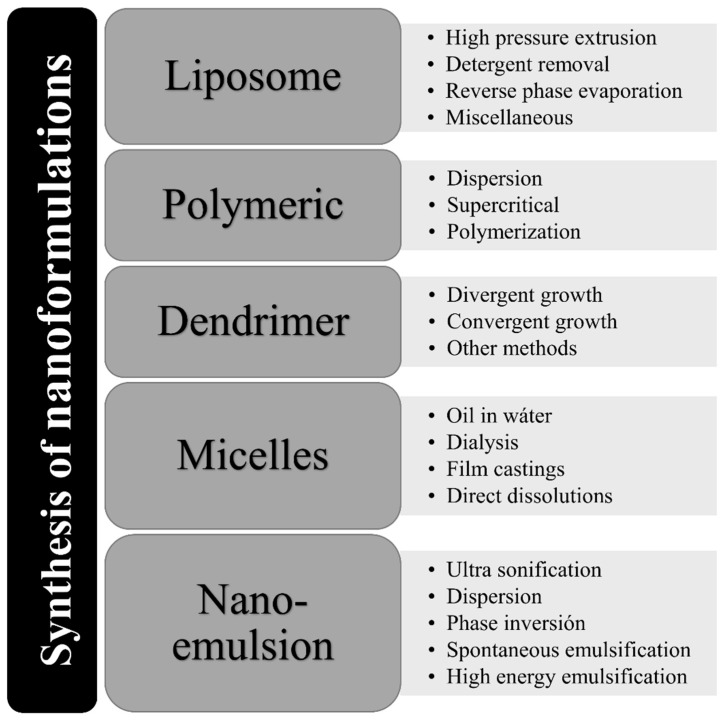
Different types of synthesis of nanoformulations.

The fate of the nanoparticle depends on the type of the methods followed for its synthesis (Figure 4). Each method produces different types of nanoparticle possessing distinct physicochemical properties. In most of the cases, nanoparticle with a size of 10–200 nm is of great interest in the medicinal applications. Different shape and size ranging from spherical to other forms having positive or negative charge on the surface of nanoparticle can be administered for toxic level in cells.

### 3.6. Cardiovascular Effects of Curcumin-Loaded Nanoparticles

CUR possess anti-inflammatory, antioxidant, and anticancer properties. Furthermore, it has been reported that this compound may protect against myocardial injury and preserve cardiac function [8]. However, its application as treatment has been hindered due to its intrinsic characteristics, such as low bioavailability, high rate of degradation, and low solubility in aqueous medium [91,99]. For these reasons, in recent years there has been an increasing interest for the development of CUR-loaded nanoformulations to overcome its pharmacokinetic limitations, which would permit to administer the compound as therapeutic agent for CVD [96,108].

In this regard, cardioprotective effects of a CUR nanoformulation in a cell model of doxorubicin-induced cardiotoxicity were explored by Carlson, et al. [192]. These authors co-loaded CUR and resveratrol at a molar ratio of 5:1 in Pluronic^®^ F127 micelles (Cur-Res-mP127). The size of Cur-Res-mP127 was 25.05 ± 0.539 nm with a PDI of 0.059 ± 0.018; interestingly, the encapsulation allowed 1617-fold the aqueous solubility of CUR with respect drug alone. The experimental approach showed that Cur-Res-mP127 reduced apoptosis and ROS in rat embryonic cardiomyocytes (H9C2) treated with doxorubicin hydrochloride, indicating cardioprotection.

On the other hand, increases in intracellular Ca^2+^ and ROS production mediated by L-type Ca^2+^ channel are major mediators of ischemia-reperfusion injury, a severe CVD. Thus, therapeutic efficacy of CUR encapsulated in poly (glycidyl methacrylate) nanoparticles alone (Cur-PGMA) and in combination with a peptide against the α-interacting domain of L-type Ca^2+^ channel (Cur-AID-PGMA) was evaluated in rat hearts exposed to ischemia-reperfusion [193]. Cur-AID-PGMA had an average diameter of 152 nm and a PDI of 0.062, with a CUR loading efficiency of 11.8% (w/w). Both Cur-PGMA and Cur-AID-PGMA exhibited beneficial effects against oxidative stress and myocardial injury following ischemia-reperfusion, suggesting that the formulations could possess therapeutic usefulness. In line with this, a study conducted by Ray, et al. [194] demonstrated that the encapsulation of CUR in carboxymethyl chitosan nanoparticles (Cur-CMC) increased its bioavailability, maintaining its bioactivity. In that report, the authors demonstrated that Cur-CMC produced regression of cardiac hypertrophy in a rat model. Likewise, nanoformulation allowed to observe beneficial effects at a low dose (5 mg/kg body weight) compared to free CUR (5 mg/kg body weight).

Hypertension may progress into more dangerous CVD, such as stroke and myocardial infarction. Since CUR possesses antihypertensive activity, Rachmawati, et al. [195] studied whether encapsulation of CUR in a nanoemulsion (Cur-NE) improve this activity in in vitro assays. The authors employed glyceryl monooleate as oil phase because this is more suitable in spontaneous nanoemulsification. Cur-NE had an average diameter of 42.93 ± 29.85 nm and a PDI of 0.36 ± 0.04, with a spherical morphology. Results showed that Cur-NE possesses higher inhibition rate of angiotensin converting enzyme with respect to pure CUR, suggesting an improvement of antihypertensive effect of the compound. 

On the other hand, due to that LV diastolic dysfunction and myocardial apoptosis are correlated, the cardiac function can be improved by sufficient control of myocardial apoptosis [141]. Concerning this, Li, et al. [196] developed CUR-loaded polyethylene glycol methyl ether-block-poly(D,L lactide) nanoparticles (Cur-PEG-PDLLA) and evaluated their effect in cardiomyocyte apoptosis induced by palmitate exposure. Authors observed that Cur-PEG-PDLLA reduced cardiomyocyte apoptosis; besides, they reported a reduction in Bax, which plays a key role in mitochondrion-mediated apoptosis, and an increment in Bcl-2, which is an antiapoptotic protein that inhibits the oligomerization of Bax. Therefore, authors suggested that the cardioprotective effect of Cur-PEG-PDLLA could be related to the regularization of the Bcl-2/Bax ratio. Interestingly, reduction of ROS production was also observed in the cardiomyocytes treated with Cur-PEG-PDLLA. A subsequent study suggested that these effects could be mediated by activation of AMP-activated protein kinase signaling pathway and regulating the expression of downstream specific proteins [197].

In 2017, Namdari and Eatemadi [198] demonstrated the cardioprotective effect of CUR-loaded magnetic hydrogel nanocomposite (Cur-NIPAAM-MAA-NP) against doxorubicin-induced cardiac toxicity in rats. They reported that the nanoparticles were successfully synthetized with 91% of efficiency of entrapment. To evaluate the cardioprotective effect of this nanoformulation, they used H9C2 cell lines (myoblastic cells); these cells were treated with free CUR and Cur-NIPAAM-MAA-NP during 72 h. The authors analyzed the expression of three heart failure markers ANP, BNP, and b-MHC genes. The decreasing of these markers suggested that Cur-NIPAAM-MAA-NP possess cardioprotective activity.

In other study, Nabofa, et al. [199] reported the elaboration of non-toxic CUR and nisin (antimicrobial peptide) based poly lactic acid nanoparticles (CurNisNp). They evaluated the protective effect CurNisNp as pretreatment in guinea pigs with isoproterenol induced myocardial necrosis. They used two doses of CurNisNp (10 and 21 mg/kg) and demonstrated that the pretreatment with CurNisNp prevented the increment in hypertrophy index observed in guinea pigs without pretreatment (control group). The authors proposed that the ability of CUR to enhance antioxidant and reduce ROS concentration is the mechanism through the CurNisNp prevents cardiac tissue damage.

Similarly, Boarescu, et al. [200] evaluated the effects of pretreatment with CUR nanoparticles (CCNP) and with free CUR on isoproterenol induced myocardial infarction in rats. CUR and CCNP were administered in three different doses (100, 150, and 200 mg/kg) for 15 days. The authors induced the myocardial infarction in the 13th day of the study. At the end of the study, blood samples were collected, and different enzymes (CK and CK-MB and oxidative stress parameters were evaluated to analyze the cardio protective, antioxidant, and anti-inflammatory effects of the CCNP. They reported a prevention of CK-MB leakage form cardiomyocytes with all the doses of CUR and CCNP, which suggested a cardioprotective effect. In addition, rats under pretreatment did not show an increment in serum levels of TNF-α, IL-6, IL-1α, IL-1β, MCP-1, unlike the control group that presented major levels of these oxidative stress parameters after the induction of myocardial infarction.

### 3.7. Curcumin Nanoformulations for Cardiovascular Effects

CUR nanoformulation can improve circulation and enhance permeation retention effect of the loaded therapeutic molecule and this is one of the most important factors in drug delivery systems [201,202]. Pre-clinical and clinical trials of various therapeutic nanoformulations have been under consideration. This may include paclitaxel albumin based nanoformulations, doxorubicin liposome nanoformulation, Paclitaxel micelle based nanoformulations, siRNA based nanoformulation and docetaxel nanoformulation [203]. 

## 4. Conclusions and Perspectives

CVD are an important cause of human deaths worldwide. New alternative therapies for CVD arise from ongoing research in the whole world. Pre-clinical and clinical studies have demonstrated the effects of CUR in CVD through its anti-hypercholesterolemic and anti-atherosclerotic effects and its protective properties against cardiac ischemia and reperfusion. These effects are scientifically verified showing CUR as a potential therapeutic candidate for CVD treatment. However, in clinical trials, a wide range of doses of CUR (20–4,000 mg) have shown different effects on CV parameters. One of the challenges for the use of CUR as a therapeutic drug is to improve its bioavailability. CUR nanomedicine formulations try to solve this obstacle by improving the CUR targeting, pharmacokinetics, efficacy, and cellular uptake. CUR nanoformulations are a therapeutic alternative in a new discovery phase. Future studies need to develop new CUR nanomedicine formulations and tested it in well-designed clinical studies.
